# Interplay between RNA interference and heat shock response systems in *Drosophila melanogaster*

**DOI:** 10.1098/rsob.160224

**Published:** 2016-10-19

**Authors:** S. Yu Funikov, S. S. Ryazansky, A. A. Kanapin, M. D. Logacheva, A. A. Penin, A. V. Snezhkina, V. Yu. Shilova, D. G. Garbuz, M. B. Evgen'ev, O. G. Zatsepina

**Affiliations:** 1 Engelhardt Institute of Molecular Biology, Russian Academy of Sciences, Moscow 119991, Russian Federation; 2 Institute of Molecular Genetics, Russian Academy of Sciences, Moscow 123182, Russian Federation; 3 University of Oxford, Oxford, UK; 4 Lomonosov Moscow State University, Moscow 119991, Russian Federation; 5 Institute for Information Transmission Problems of the Russian Academy of Sciences, Moscow 127051, Russian Federation

**Keywords:** *Drosophila melanogaster*, heat shock, microRNA, *hsp70*

## Abstract

The genome expression pattern is strongly modified during the heat shock response (HSR) to form an adaptive state. This may be partly achieved by modulating microRNA levels that control the expression of a great number of genes that are embedded within the gene circuitry. Here, we investigated the cross-talk between two highly conserved and universal house-keeping systems, the HSR and microRNA machinery, in *Drosophila melanogaster*. We demonstrated that pronounced interstrain differences in the microRNA levels are alleviated after heat shock (HS) to form a uniform microRNA pattern. However, individual strains exhibit different patterns of microRNA expression during the course of recovery. Importantly, HS-regulated microRNAs may target functionally similar HS-responsive genes involved in the HSR. Despite the observed general downregulation of primary microRNA precursor expression as well as core microRNA pathway genes after HS, the levels of many mature microRNAs are upregulated. This indicates that the regulation of miRNA expression after HS occurs at transcriptional and post-transcriptional levels. It was also shown that deletion of all *hsp70* genes had no significant effect on microRNA biogenesis but might influence the dynamics of microRNA expression during the HSR.

## 1. Introduction

All living organisms depend on the interaction of a complex set of biochemical reactions directed at maintaining the dynamic homeostatic balance in cells [1]. However, even short-term drastic environmental changes such as temperature elevation (i.e. heat shock, HS) force the cells to adapt by adjusting the basic gene expression profile [1–3]. One of the mechanisms by which it is possible to achieve rapid modulation of cellular metabolism is the regulation of the microRNA (miRNA) expression and function.

miRNAs are a class of small non-coding RNAs approximately 21–23 nucleotides in length that originate from RNA polymerase II (Pol II) produced transcripts to form a stem-loop structure via digestion with RNase III enzymes, including the Drosha/DGCR8 complex in the nucleus and Dicer in the cytoplasm [4,5]. Processed or mature miRNAs bind to Argonaute (Ago) proteins and form the RNA-induced silencing complex (RISC), which is capable of recognizing complementary sites in the target mRNA located primarily in the 3′ untranslated regions [4,5]. As miRNAs control protein synthesis by promoting either target mRNA degradation or translation inhibition, they play essential roles in various vital processes, including cell proliferation, differentiation, development and senescence [4,6,7].

The regulatory potential of miRNAs is enormous. That is, more than 60% of protein-coding genes are predicted as targets for miRNA regulation [8]. Generally, one miRNA targets a set of mRNAs, and one mRNA may represent a potential target for multiple miRNAs. Owing to this property, miRNAs and their targets form a complex regulatory network [9,10]. When cells undergo metabolic imbalance upon encountering stress stimuli, miRNAs serve as a convenient molecular tool for either tuning or re-programming the genome expression pattern [11,12]. A number of studies have demonstrated that stress, in particular HS, can alter the expression level of miRNAs and affect RISC function [13–18]. Genes involved in miRNA-mediated silencing can themselves be targets for regulation upon stress. For example, mRNA translational repression of cationic amino acid transporter 1 (CAT-1) by miR-122 is relieved upon exposure to a stress such as amino acid starvation [19]. Later, it was shown that modification of Ago2 by poly(ADP-ribose) increases during stress and results in the relief of a subset of mRNAs from miRNA-mediated translational repression in human cells [20]. It was also shown that p38 mitogen-activated protein kinase (MAPK) directly phosphorylates Drosha and reduces its interaction with DGCR8 (Pasha in *Drosophila*) under stress conditions [21]. Thus, one may conclude that a general reduction in the efficacy of miRNA-mediated silencing occurs during stress exposure. However, the mechanisms underlying the regulation of miRNA expression during the HSR are poorly defined. In particular, it is unclear how miRNA contributions modulate the formation of an adaptive gene expression profile.

Stress stimuli, especially severe HS, induce the phosphorylation of eukaryotic initiation factor 2α (eIF2α) and lead to a shutdown of protein synthesis at the stage of translation initiation [22,23]. Several studies have shown that HS-induced accumulation and decay rates of Hsp70 positively correlate with the rate of recovery of normal-functioning translation machinery after stress termination [23–25]. The important question in this context is how the dynamics of this recovery of HS-repressed protein synthesis contribute to miRNA expression modulation and whether there are differences in the miRNA expression pattern after severe HS in strain lacking *hsp70* genes.

Herein, we analysed deep-sequencing data of miRNA expression upon severe HS in the process of chaperone-driven recovery of normal protein synthesis in several *Drosophila melanogaster* strains. We also investigated the possible participation of the major stress protein Hsp70 in the modulation of the miRNA expression pattern in *D. melanogaster* under stress conditions*.* We performed this analysis using a *D. melanogaster ‘hsp70^−^* strain’ that lacks all *hsp70* genes [26]. Recently, using the same model system, we analysed the deep-sequencing data of piRNA expression after HS treatment and demonstrated that HS results in strain-specific expression modulation of certain double-stranded piRNA clusters [27].

We explored various bioinformatics approaches to analyse the obtained deep-sequencing data of miRNA expression during the HSR and demonstrated that the expression levels of miRNAs in the compared *D. melanogaster* strains exhibit significantly more differences under control conditions (25°C) than after severe HS. After stress, miRNA expression tends to form a similar expression pattern in all *Drosophila* strains investigated so far, including strains lacking *hsp70* genes. We also performed a gene ontology (GO) analysis of potential targets of HS-responsive miRNAs and compared our results with known HS transcriptome data to determine the degree to which the observed miRNA expression modulation correlates with transcription levels of corresponding HS-regulated targets during the HSR.

## 2. Results

### 2.1. Translational patterns after heat shock are strikingly different in the *hsp70^−^* strain and control strains

We used three *D. melanogaster* strains in our experiments, including a strain carrying a deletion of all *hsp70* genes (*hsp70^−^* strain) [26]. The two other strains contain a normal set of *hsp70* genes and include *w^1118^*, which is an ancestor of the *hsp70^−^* strain, and an unrelated strain *yw*. The heat treatment conditions and recovery intervals after HS were empirically established based on the physiological characteristics of the response of *Drosophila* cells to severe HS [23,24,28].

**Figure 1.**
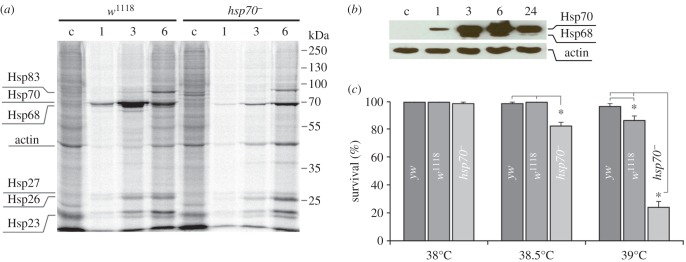
The peculiarities of the HSR in the studied *D. melanogaster* strains. (*a*) Incorporation of [^35^S]-methionine in *Drosophila* salivary gland proteins in the *w^1118^* and *hsp70^−^* strains indicates diverse recovery rates of original protein synthesis levels between the compared strains. Although strong induction of Hsp70 served as the major indicator of the HSR in the control *w^1118^* strain, Hsp70 was not synthesized in the *hsp70^−^* strain, as expected; however, Hsp68 and a group of small Hsps were induced. Six hours after HS, the *w^1118^* strain exhibited the original level of total protein synthesis, whereas protein synthesis was not fully recovered in the strain lacking *hsp70* genes. Equal amounts of proteins were loaded in all lanes. (*b*) Western blot of protein extracts from the *w^1118^* strain shows Hsp70 levels at the indicated times of recovery after HS treatment (38.5°C for 30 min). (*c*) The survival of adult y*w*, *w^1118^* and *hsp70^−^* flies after severe HS exposure. The flies of the *w^1118^* strain exhibited reduced survival compared with the *yw* strain (86% and 96%, respectively, *p* < 0.05) when subjected to a higher temperature (39°C), indicating that flies of the *yw* strain have greater thermoresistance. c, control conditions (25°C); 1, 3, 6 and 24 indicate the recovery time (hours) after HS exposure termination. *p ≤ 0.05; the error bars represent the standard deviation.

In our experiments, short-term severe HS (38.5°C for 30 min) in *D. melanogaster* led to an almost complete temporal shutdown of the de novo synthesis of [^35^S]-labelled Met proteins in larval salivary glands and the induction of heat shock protein (Hsp) synthesis immediately after HS termination (figure 1*a*). After a 6 h recovery period, the original protein levels were mostly restored in the strains with the normal set of *hsp70* genes; in contrast, the synthesis of most cellular proteins was still strongly inhibited at this time point in the *hsp70^−^* strain (figure 1*a*), confirming the crucial role of Hsp70 in protein synthesis restoration, as was previously demonstrated [23,24,28]. Notably, maximal Hsp70 synthesis was achieved 6 h after HS in the control strain (figure 1*b*). Based on these results, we considered this time interval (6 h after HS) as a pivotal time point that allows the miRNA expression pattern to be compared between strains, with specific attention on the *hsp70^−^* strain in the context of the strikingly different protein levels.

Thermotolerance experiments demonstrated that the temperature treatment used (38.5°C for 30 min) represents a survival threshold for the *hsp70^−^* strain. The flies from this strain showed significantly reduced survival (approx. 80%, *p* < 0.05) after HS exposure, whereas the survival values for the other strains with a normal set of *hsp70* genes were nearly 100% after this treatment (figure 1*c*). Based on these observations, we proposed that if the cellular activity of Hsp70 somehow affects miRNA biogenesis, then the expected changes in the miRNA expression pattern should be more drastic after the severe HS implemented in the aforementioned experiments.

Therefore, taking into account all these results, we sequenced small RNA libraries obtained from adult females of the three *D. melanogaster* strains after HS treatment. The samples for comparison were prepared after 1, 6 and 24 h HS recovery periods to examine the kinetics of miRNA expression modulation during the HSR.

### 2.2. *Drosophila melanogaster* strains exhibit significant differences in miRNA levels under normal conditions that are alleviated by heat shock

To identify the miRNAs with significant changes in expression after HS exposure, we set a threshold and only considered expression changes no less than log_2_FC ≥ 1.5. Furthermore, we discarded miRNAs with less than 50 counts as lowly expressed. We also performed correlation analysis and principal component analysis (PCA), which showed a high degree of miRNA expression similarity between biological replicates (electronic supplementary material, figure S1). Initially, we performed a pairwise analysis of the differential expression of miRNAs between the strains at all of the time intervals after HS and identified 73 (*P*_adj_ ≤ 0.05) differentially expressed miRNAs (electronic supplementary material, figure S2).

**Figure 2.**
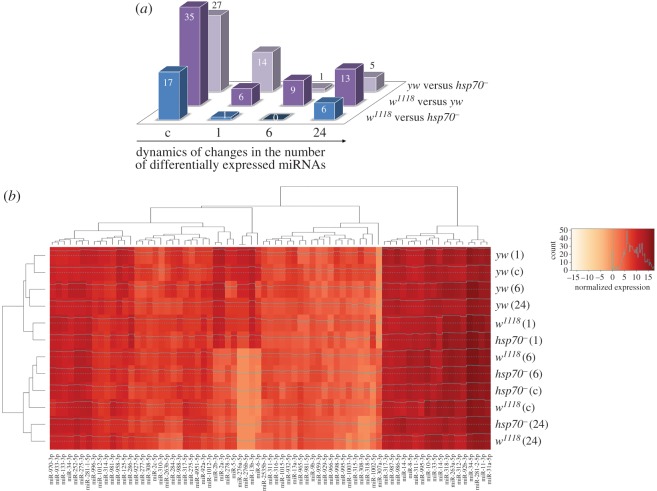
miRNA expression is adjusted to similar levels after HS treatment in all of the studied *D. melanogaster* strains. (*a*) The interstrain differences in the miRNA expression levels among all of the studied strains were substantially reduced after HS and tended to recover at 24 h. Six hours after HS, miRNAs exhibited an almost identical expression pattern in the closely related strains *w^1118^* and *hsp70^−^*; a rather similar pattern was observed in these strains and the unrelated (*yw*) strain. (*b*) Heat map of the expression values of 73 differentially expressed miRNAs. It is evident that although the miRNA expression profiles in the studied *D. melanogaster* strains are different under normal conditions, they all tended to form a similar expression profile 1 h after HS. The miRNA expression pattern of the *yw* strain in response to HS did not differ drastically during recovery; hence, this strain is more stable with regard to miRNA synthesis. ‘Count’ indicates the number of miRNAs expressed within a particular range of normalized expression values. c, control conditions (25°C); 1, 6 and 24 indicate the recovery time (hours) after termination of HS exposure.

We divided the HS-responsive miRNAs into two classes (electronic supplementary material, figure S2). The first class represents differentially expressed miRNAs that were detected when comparing the strains under normal physiological conditions (25°C). We demonstrated that the interstrain differences in the miRNA expression of class #1 miRNAs were alleviated after HS, resulting in a highly similar miRNA expression pattern in all of the studied strains (figure 2*a*; electronic supplementary material, S2). Notably, these changes represent different fluctuations in miRNA expression (upregulation or downregulation) in each strain.

The second class comprises miRNAs that are characterized by similar levels under normal conditions but exhibit significant changes over the course of the HSR (electronic supplementary material, figure S2). Similar to the class #1 miRNAs, class #2 miRNAs gradually tended to return to their original strain-specific expression levels over the course of the recovery period after HS. Notably, in the closely related strains *w^1118^* and *hsp70^−^*, miRNAs belonging to both classes exhibited fewer differences in miRNA expression levels compared with the unrelated *yw* strain (electronic supplementary material, figure S2).

As expected, the expression patterns of HS-responsive miRNAs of closely related strains (*w^1118^* and *hsp70^−^*) were clustered together at almost all of the time intervals and were not clustered with the unrelated *yw* strain (figure 2*b*). However, 1 h after HS, the miRNA expression patterns in the *w^1118^* and *hsp70^−^* strains exhibited more similarity to the *yw* strain miRNA expression pattern rather than with their own at other time intervals (figure 2*b*). It is important to note that, because all 73 HS-responsive miRNAs were included in this analysis, both of the identified classes of differentially expressed miRNAs described above are apparently involved in the formation of a uniform miRNA expression pattern after HS.

Initially, we expected that deletion of all of the *hsp70* genes would have a more pronounced impact on miRNA expression after HS than interstrain polymorphisms. The accumulated data demonstrated that miRNAs may change their expression level after HS (both upregulation and downregulation) depending on their original strain-specific level.

### 2.3. Different groups of miRNAs that are levelled by heat shock regulate functionally similar genes during the heat shock response

**Figure 3.**
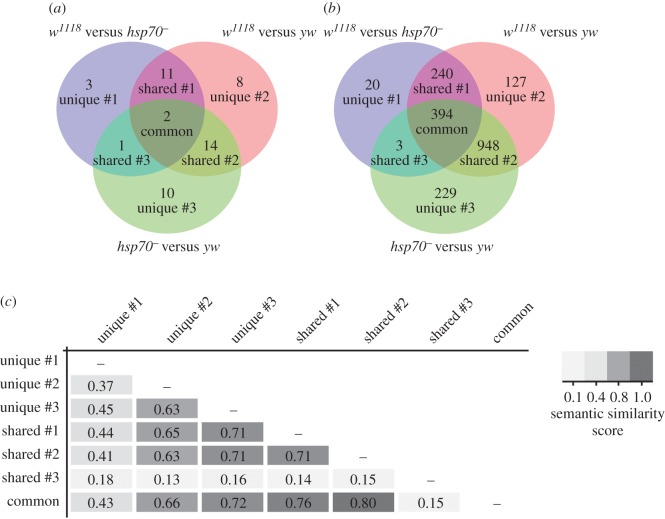
Analysis of the predicted target genes of levelled miRNAs (class #1 miRNAs). (*a*) Venn diagram of all of the miRNAs belonging to class #1. For a description of the defined miRNA classes, see the text. (*b*) Venn diagram of all of the predicted target genes in the corresponding groups. (*c*) Analysis of semantic similarity of the enriched GO terms in all of the levelled miRNA groups (*p* ≤ 0.05).

Most of the levelled miRNAs exhibited interstrain expression polymorphism under normal conditions (electronic supplementary material, figure S2). Although the levelling effect after HS was observed in all of the studied strains, the nature of the affected miRNAs varied significantly among the strains (figure 3*a*). Therefore, we defined ‘common’ miRNAs as those levelled by HS in all strains, ‘shared’ miRNAs as those shared by any two strains used and, finally, ‘unique’ miRNAs as those levelled in only one strain (figure 3*a*; electronic supplementary material, table S2). We propose that, despite the disparate sets of levelled miRNAs, they are involved in the regulation of similar biological processes during the course of the HSR.

To test this hypothesis, we tested the similarity of the GO classification with regard to the predicted targets of the HS-responsive levelled miRNA belonging to class #1. The final list of target genes was not overly large for unique group #1 and shared group #3, in which 20 and three genes were regulated, respectively (figure 3*b*). In contrast, the other groups comprised a large number of targets; a common group of levelled miRNAs (miR-286-3p and miR-5-5p) may target 394 genes involved in most of the cellular processes regulated by levelled miRNAs (figure 3*b*; electronic supplementary material, S3). Importantly, different groups of levelled miRNAs may target genes with the same function. For instance, the target genes for all three unique groups, shared groups #1 and #2, and the common group are involved in locomotion, cell migration, transcription regulation and signal transduction processes (electronic supplementary material, figure S3).

We also performed an analysis of the semantic similarity of the enriched top GO terms (i.e. those having a non-adjusted nominal *p* ≤ 0.05) among all of the groups of levelled miRNAs (figure 3*c*). The results show that the largest groups of levelled miRNAs (unique #2, unique #3, shared #1, shared #2 and common) have highly similar top GO terms (mean semantic similarity score = 0.7; *p* ≤ 0.05) with regard to their mRNA targets (figure 3*c*).

In summary, these data indicate that different sets of levelled miRNAs regulate a highly similar set of mRNA targets during the HSR. Thus, the observed levelling effect of miRNA expression after HS probably represents a species-specific feature that functions during the fine-tuning of the cellular homeostatic processes during the HSR.

### 2.4. Individual miRNAs exhibit different expression profiles during the course of the heat shock response

**Figure 4.**
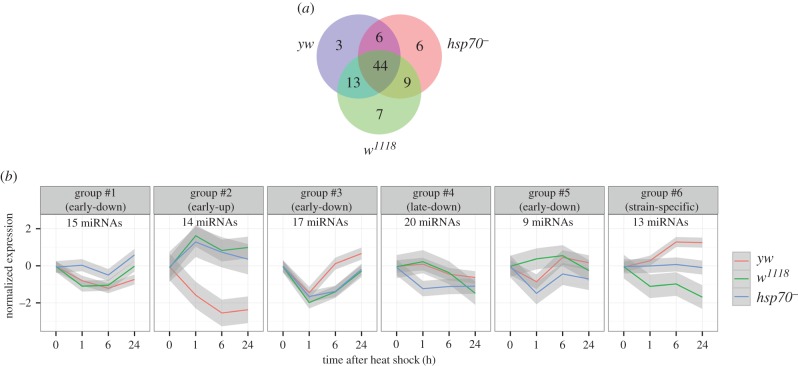
Time-course analysis of the miRNA expression profiles after HS. (*a*) Venn diagram of 88 miRNAs exhibiting significant changes in their expression profiles after HS. (*b*) Averaged expression profiles of 88 miRNAs with significantly altered expression profiles clustered based on the level of their similarity (hierarchal clustering, ‘complete’ method). The averaged expression profiles for each cluster were obtained by fitting to a linear regression model. The grey zones around the lines depict the 95% confidence intervals.

To further analyse both the similarities and differences in specific miRNA content at different time intervals after HS, we performed a time-course analysis of miRNA expression. This analysis detected 88 miRNAs whose expression profiles were significantly modulated after HS (figure 4*a*; electronic supplementary material, table S3). Of these, half (44 miRNAs) exhibited profile changes after HS in all three strains. Few (28) exhibited changes in their expression profile in two strains, and a smaller number (16) exhibited changes in their expression in only one strain (figure 4*a*).

The cluster analysis identified six groups of miRNAs (figure 4*b*; electronic supplementary material, table S3). The expression levels of miRNAs belonging to group #1 were downregulated in the *yw* and *w^1118^* strains at 1 h, whereas the same miRNAs were downregulated in the *hsp70^−^* strain 6 h after HS. Similar expression trends (downregulation after HS) were characteristic of the miRNAs belonging to groups #3 and #5 (figure 4*b*). Thus, these groups of miRNAs can be referred to as ‘early-down’.

The ‘early-up’ signature (i.e. upregulation 1 h after HS) was exhibited by miRNAs belonging to group #2 in the *w^1118^* and *hsp70^−^* strains (figure 4*b*). The miRNAs belonging to group #4 were gradually downregulated after a 24 h recovery period and were annotated as having a ‘late-down’ signature (figure 4*b*).

Notably, the miRNA expression profiles of several groups demonstrate a strain-specific response: in the *w^1118^* strain, miRNAs belonging to group #5 showed either modest upregulation or steady-state levels compared with the other strains; in the *yw* strain, miRNAs belonging to group #2 showed the opposite trend in expression dynamics when compared with the *w^1118^* and *hsp70^−^* strains. Finally, group #6 contained miRNAs demonstrating a completely different expression profile: upregulation in the *yw* strain, downregulation in the *w^1118^* strain and steady-state levels in the *hsp70^−^* strain (figure 4*b*).

The strain-specific expression profiles of miRNAs probably demonstrate the previously described levelling effect of miRNA expression during the HSR. Indeed, comparison of miRNAs from group #6 (strain-specific) with the list of levelled miRNAs belonging to the previously described class #1 showed that 10 of 13 miRNAs were shared between them (see electronic supplementary material, tables S2 and S3). The other miRNA groups have approximately the same number of shared and different miRNAs with class #1 and class #2 (electronic supplementary material, tables S2 and S3).

At 6 h after HS termination, the miRNAs in almost all strains (with exception of the #2 and #6 groups) exhibited similar expression profiles (figure 4*b*). Thus, the HS-mediated regulation of miRNA expression is probably independent of the recovery of protein synthesis, which is strikingly different in the *hsp70^−^* strain and the *w^1118^* strain at the 6 h time point after HS (figure 1*a*). However, the expression of miRNAs after HS cannot be referred to as an Hsp70-independent process, because the miRNAs in group #1 and group #4 in the *hsp70^−^* strain exhibited different expression profiles than the same miRNAs in the *w^1118^* and *yw* strains (figure 4*b*).

A significant number of miRNAs in the *Drosophila* genome are encoded as genomic miRNA clusters. Our analysis concluded that most of the clustered HS-responsive miRNAs demonstrate a similar unidirectional expression profile that is predominantly downregulated after HS. These are clusters 13b–2c, 310–313, 994–318, 2a–2b and 6–309 based on their genome organization (electronic supplementary material, table S3). However, the expression levels of other clustered miRNAs respond to HS in an individual manner and independently of each other or even demonstrate opposing expression trends. A good example of such a response is represented by the expression profiles of miRNAs belonging to cluster 277–34 (figure 4*b*; electronic supplementary material, table S3). Although miR-277 is upregulated, miR-34 is downregulated and miR-317 is steadily expressed after HS in the *w^1118^* strain. The same pattern is observed in cluster 100–125 (let-7-5p, a highly expressed guide strand that is not HS-modulated) and 314–956 (miR-314 is steadily expressed, whereas miR-956 is downregulated; figure 4*b*; electronic supplementary material, table S3).

### 2.5. Heat shock decreases the levels of pri-miRNAs and downregulates most core miRNA pathway genes

**Figure 5.**
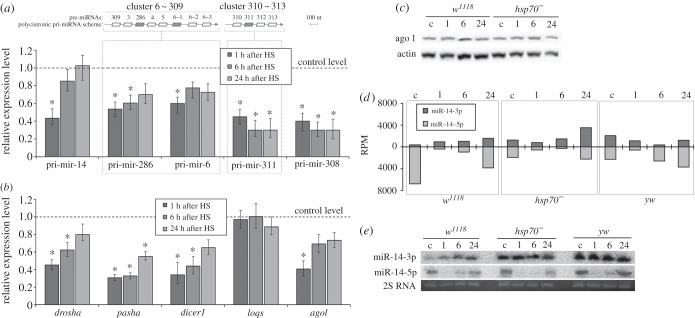
The peculiarities of the regulation of miRNAs, their precursors and components of miRNA expression machinery after severe HS. (*a*) Relative expression levels of primary pri-miRNAs of candidate HS-responsive mature miRNAs in the *w^1118^* strain at different time intervals after HS as determined using qPCR. In the schematic of a polycistronic transcript of a miRNA, grey parallelograms represent the analysed pri-miRNA region and the arrowheads indicate the 3′-end of the transcript. (*b*) Relative expression levels of miRNA biogenesis pathway genes after HS in the *w^1118^* strain. *p ≤ 0.05 versus control mRNA levels; the mean values ± error bars for three replicates are represented in (*a*) and (*b*). (*c*) Western blot analysis of Ago1 in the *w^1118^* and *hsp70^−^* strains. (*d*) Number of reads of miR-14-3p and miR-14-5p at different time intervals after HS. (*e*) Northern blot of miR-14-3p and miR-14-5p after HS. The 30-nucleotide 2S rRNA band visualized by ethidium bromide staining on the polyacrylamide gel before transfer of the RNA to the blotting membrane was used as a loading control. c, control conditions (25°C); 1, 3, 6 and 24 indicate the recovery time (hours) after termination of HS exposure.

The regulation of mature miRNA content may be due to either increased or decreased transcription levels of the corresponding pri-miRNAs. Using qPCR, we found that HS exposure led to a decrease in the expression (two- to 2.5-fold, *p* ≤ 0.05) of several selected pri-miRNAs, specifically, pri-mir-14, pri-mir-6, pri-mir-286, pri-mir-311 and pri-mir-308 in the *w^1118^* strain (figure 5*a*). After a 24 h recovery period, pri-mir-14, pri-mir-6 and pri-mir-286 gradually returned to their original levels. Surprisingly, although pri-mir-14 and pri-mir-308 were downregulated after HS, mature miR-14-3p and miR-308-5p were upregulated (figures 5*a* and 4*b*; electronic supplementary material, table S3). The same applies to miR-6 and miR-286, which are encoded in the genome as miRNA cluster 6–309 and transcribed as a single pri-miRNA transcript. As qPCR experiments showed, the levels of pri-mir-6 and pri-mir-286 decreased after HS, apparently owing to downregulation of the entire 6–309 cluster. However, mature miR-5-5p, miR-286-3p and miR-6-3p were concurrently upregulated after HS. In contrast, miRNAs originating from cluster 310 to 313 exhibited highly similar expression profiles, and their decreased levels of expression correlate with the downregulation of pri-mir-311 (figures 5*a* and 4*b*; electronic supplementary material, table S3).

It was recently demonstrated that the level of Drosha is decreased after HS in human cells [21]. However, in *Drosophila,* the downregulation of *drosha* and other genes in the miRNA biogenesis pathway were not detected after mild heat treatment [2]. We demonstrated that severe HS results in the downregulation of core miRNA pathway genes, specifically *dicer1*, *drosha*, *ago1* and *pasha,* in all of the studied strains (figure 5*b*; electronic supplementary material, S4). However, *loqs*, a partner of Dicer1, represents a prominent exception to the rule, as its mRNA level was stable during the HSR. Importantly, the expression of the miRNA biogenesis pathway genes often returned to the original level 24 h after HS. Notably, despite the apparent mRNA downregulation, the Ago1 protein levels remained steady after HS in the *w^1118^* and *hsp70^−^* strains (figure 5*c*).

The alteration of miRNA expression may also occur owing to an ‘arm-switching’ mechanism [29]. Based on this premise, we observed the HS-induced ‘arm-switching’ of miR-14. Surprisingly, this event was strain-specific. One hour after HS, miR-14-5p was downregulated in all of the studied strains and was restored to its original level at 24 h, whereas miR-14-3p expression remained stable in the *hsp70^−^* and *yw* strains; however, in the *w^1118^* strain, miR-14-3p was upregulated (figure 5*d*,*e*).

RNA editing in the ‘seed’ regions of miRNAs can lead to the production of modified miRNAs that can potentially recognize different sets of targets than unmodified miRNAs [30]. Thus, HS-mediated expression changes in edited miRNAs may compromise miRNA target prediction analysis. We evaluated our miRNA libraries for the A–G and T–C types of miRNA editing, and found that miR-1, miR-9c, miR-281, miR-274, miR-999 and miR-971 undergo editing at certain time intervals in a strain-specific manner (electronic supplementary material, table S4). Notably, the modified miRNAs do not exhibit a specific HS-induced pattern (i.e. up- or downregulation) after HS. Moreover, none of the miRNA editing sites were located in the ‘seed’ region. Therefore, we conclude that the impact of miRNA editing on miRNA action during the HSR is minimal if existent at all.

### 2.6. Heat shock-responsive miRNAs target genes involved in the heat shock response

Full genome analysis of gene expression at different time intervals after HS identified the early-up, early-down and late-up groups of HS-responsive genes [2]. The early-up and early-down groups of genes show alterations of gene expression in the first hours after HS exposure, whereas the last group includes genes that are upregulated 16 and 32 h after HS termination [2]. We hypothesized that HS-induced perturbations in miRNA expression may contribute to alterations in the abundance of their target mRNAs during the HSR.

To test this hypothesis, we evaluated if the HS-responsive miRNAs defined during the time-course analysis (figure 4*b*) may target stress-responsive genes. We restricted our analysis to 299 genes that represent the minimal and universal subset of genes responding to different types of stresses in different strains, as independently determined by several groups [2,31–33]. To optimize the target analysis, we combined all of the downregulated miRNAs belonging to groups #1, #3 and #5 (figure 4*b*) and redefined them as a single downregulated group.

**Figure 6.**
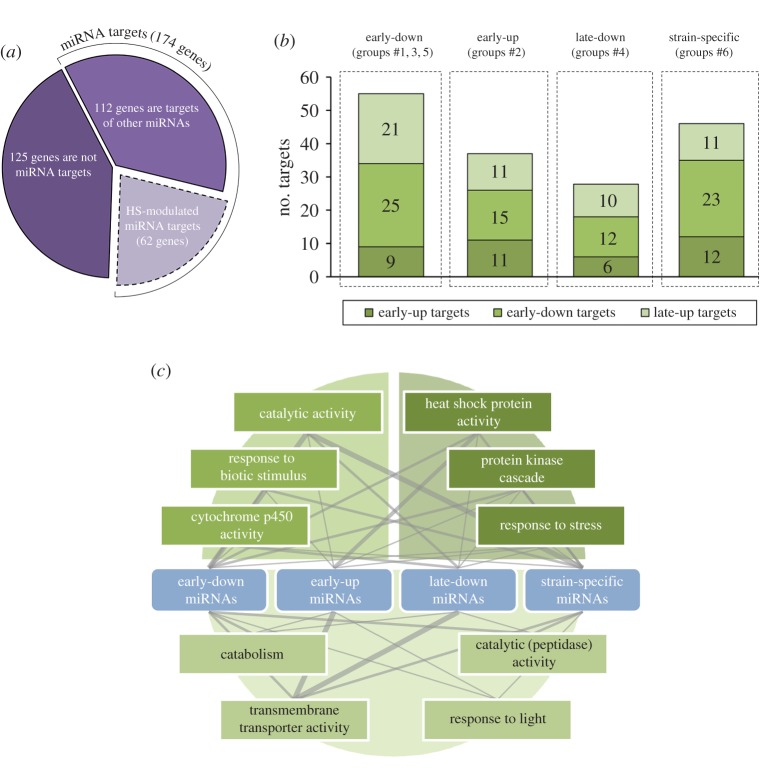
Targeting analysis of the universal set of stress-responsive genes by HS-responsive miRNA groups. (*a*) Nearly 60% (174 of 299 genes) of stress-responsive genes may be potential targets of miRNAs. Among these, 35% (62 genes) are targets of HS-responsive miRNAs. (*b*) Distribution of potential targets of the miRNA groups defined by time-course analysis among the three groups of the stress-responsive genes. (*c*) Schematic of the proposed regulatory network linking miRNA groups and their targets (in GO terms of most target-enriched categories) during the HSR. All three groups of stress-responsive genes are divided into three sectors and highlighted by colours corresponding to those marked in (*b*). The line width indicates the number of genes regulated by a certain miRNA group.

Our analysis indicated that miRNAs may target 174 of the 299 selected stress-responsive genes (figure 6*a*). Among these genes, 62 (35%) are potential targets of stress-responsive miRNAs (figure 6*b*; electronic supplementary material, table S5). In accordance with our theory regarding the impossibility of eliminating the attributes of miRNAs and their targets within the same time interval, we found that each miRNA group may target all three groups of stress-responsive genes—the early-down, early-up and late-up groups. For example, the early-down miRNAs may potentially target 25 early-down, nine early-up and 21 late-up stress-responsive genes (figure 6*b*).

The subsequent GO enrichment analysis of the identified stress-responsive targets demonstrated that the early-down, early-up, late-up and strain-specific miRNA groups very often targeted stress-responsive genes belonging to the same functional category (figure 6*c*; electronic supplementary material, table S5). For example, genes encoding HSPs are targeted mainly by the early-up, early-down and strain-specific miRNA groups, and genes providing catalytic activity were regulated by early-down, late-down and strain-specific miRNAs; genes involved in transmembrane transport were potential targets for all the studied miRNA groups (figure 6*c*). Notably, miRNAs from each considered group also had targets with specific functions that are not present in the other groups (electronic supplementary material, table S5). These findings may indicate that different miRNAs are functioning within a regulatory miRNA : mRNA network during the progression of the HSR and regulate similar cellular processes at all steps in the restoration of cellular homeostasis after stress.

## 3. Discussion

To date, a number of studies on different animal and plant species demonstrated that stress results in the modulation of miRNA levels [13–15,34,35]. A crucial role for a specific set of miRNAs during developmental survival as well as in behavioural functions such as locomotion and reproduction after HS was recently revealed in *Caenorhabditis elegans* [18]. The mode of miRNA-silencing can also be altered (predominantly decreased) during stress exposure [16,17,19,20]. The degree of miRNA modulation strongly depends on the severity and duration of stress encountered. Most of the previous studies investigated miRNA activity either directly during HS exposure or immediately after stress termination [13–17,19,20].

In this study, we investigated the dynamics of the modulation of miRNA expression during the HSR in *D. melanogaster*. For this purpose, we analysed the changes in miRNA abundance during the HSR at different time points, using deep sequencing of small RNAs from three *Drosophila* strains (the closely related *w^1118^* and *hsp70^−^* strains as well as the unrelated *yw* strain). We have shown that HS-responsive miRNAs can be separated into two classes: class #1 miRNAs with expression patterns that differed in strains under normal conditions but levelled after HS treatment, and class #2 miRNAs in which expression was significantly altered only after HS treatment. Although such separation of miRNAs into two classes is arbitrary, we propose that this classification best reflects the mode of miRNA modulation in response to severe HS. The initial interstrain polymorphisms of the expression of the first class of miRNAs reflects phenotypic interstrain differences that undergo substantial levelling after HS (figure 2*a*). We demonstrated that such levelling of miRNA levels after HS occurs owing to individual (i.e. strain-specific) adjustments of the miRNA levels to a uniformed standard.

To determine the importance of the observed HS-induced levelling effect of miRNA expression on the physiology of cells, we conducted a GO analysis of all potential targets of levelled miRNAs. This analysis indicated that, functionally, different sets of levelled miRNAs result in highly similar miRNA-mediated regulation of gene expression during the HSR (figure 3; electronic supplementary material, S3). We assume that the levelling of miRNA expression under stress conditions results in cells entering an adaptive (and probably species-specific) state, which contributes to overall survival and an efficient recovery of cells after HS. Concurrently, strain-specific expression of HS-responsive miRNAs belonging to the second class probably reflects the various pathways of recovery of cellular homeostasis in individual *Drosophila* strains after HS based on their different evolutionary history.

The mechanism of levelling of miRNA expression is thought to be sophisticated. Severe HS results in the cessation of protein synthesis via eIF2α phosphorylation, which triggers the formation of subcellular structures called stress granules (SGs) that preserve most cellular mRNAs and miRNAs from degradation during stress [36]. A significant number of mRNAs are degraded within another cytoplasmic structure called P-bodies that are enriched with enzymes responsible for the decapping, deadenylation and cleavage of mRNAs [37]. It is known that the miRNA biogenesis pathway is linked with SG and P-bodies because they are bound to miRNAs and Ago proteins [11]. It appears reasonable to speculate that the targets of levelled miRNAs coupled with miRNAs may be selectively transported to either P-bodies or SGs, which leads to the degradation or stabilization of both mRNAs and miRNAs, respectively. It is likely that the HSR includes a highly coordinated programme of preserving the same sets of miRNAs and mRNAs, which may explain the levelling effect of strain-specific differences in miRNA profiles observed after HS. However, this hypothesis is not the only possible explanation of the observed phenomenon and definitely requires further study.

To show the differences and similarities of the modulation of miRNA expression during the HSR in different *Drosophila* strains more clearly, we conducted a time-course analysis that allowed us to monitor the changes in miRNA expression over time. Based on the obtained data, we concluded that all of the *Drosophila* strains have common (and apparently) species-specific as well as strain-specific features of miRNA expression regulation during the HSR. It is now clear that despite the absence of all of the *hsp70* genes, the miRNA expression profiles of the closely related strains *w^1118^* and *hsp70*^−^ have fewer differences compared with that of the unrelated *yw* strain. The expression of miRNAs belonging to groups #2 and #3 distinguished the *yw* strain from the other two and represents a clear example of strain-specific miRNA expression after HS (figure 4*b*). Moreover, miRNA expression appears to be more robust to HS in the *yw* strain as observed by the clustering of all of the differentially expressed miRNAs (figure 2*b*). We have also shown that the *yw* strain is more thermoresistant compared with the *w^1118^* and *hsp70^−^* strains (figure 1*c*). It is possible that the increased thermoresistance of the *yw* strain may be partly explained by strain-specific properties of the miRNA-mediated HSR.

What are the possible mechanisms underlying the alteration of miRNA abundance after HS treatment? Severe HS should reduce the activity of RNA Pol II, and, as expected, we observed decreasing pri-miRNA transcriptional levels and pronounced downregulation of the core miRNA biogenesis pathway genes (figure 5*b*) [38]. This finding can explain the downregulation of a subset of miRNAs, including the clusters 310–313, 994–318, 2a–2b and 13b–2c (figure 5*a*; electronic supplementary material, table S3).

We have also demonstrated the post-transcriptional regulation of several clustered miRNAs. For example, expression levels of miRNAs derived from clusters 277–34 and 314–956 are individually modulated even in opposing manners during the HSR. Moreover, the expression level of the cluster 6–309 transcript is decreased, whereas the levels of mature miRNAs belonging to this cluster are upregulated. Such regulation may include not only cluster-derived miRNAs, but also other miRNAs. Despite the observed downregulation of pri-mir-14 and pri-mir-308, mature miRNAs (e.g. miR-14-3p and miR-308-5p) originating from these precursor molecules exhibit upregulation after HS (figure 5*a*; electronic supplementary material, table S3). Because clustered miRNAs are transcribed as single primary pri-miRNA transcripts, the observed expression pattern can be achieved only by post-transcriptional regulation of mature miRNA [39,40]. Overall, these data suggest that miRNA expression regulation after stress occurs at both the transcriptional and post-transcriptional levels.

In the course of our analysis, we found evidence that strain-specific modes of the miRNA-mediated response during the HSR may include not only differential expression of miRNAs, but also the phenomenon of arm-switching [29]. One example involves miR-14-3p, which exhibits minimal expression levels under normal conditions but is actively accumulated upon HS treatment in the *w^1118^* strain (figure 5*d*,*e*). Because miR-14-3p regulates lipid and insulin metabolism and suppresses cell death, we speculate that its involvement in the regulation of these processes is crucial especially during the HSR but may significantly differ in different strains under normal physiological conditions [41,42].

The detected changes in miRNA abundance after HS treatment may have an impact on the transcriptome and proteome of the cell. To predict the consequences of miRNA expression changes in more detail, we conducted a target analysis of HS-responsive miRNAs. For this purpose, we used a minimal universal set of stress-responsive genes reported by different groups [2,31–33]. However, it is clear that this type of analysis has obvious limitations. For instance, the response of target mRNA expression to changes in miRNA expression is usually delayed temporally [43,44]. As a result, the alteration of miRNAs and the abundance of their corresponding mRNA targets should occur in different time intervals. In addition, these stress-responsive genes were identified only by analysis of transcriptome, which underestimates the number of miRNA targets. However, we believe that this approach may be applied to evaluate the nature of the impact of miRNA-mediated silencing on the physiology of cellular response to stress exposure.

In the course of this analysis, we demonstrated that approximately 20% of the stress-responsive genes represent potential targets of HS-responsive miRNAs (figure 6*a*). Generally speaking, HS-responsive miRNAs may regulate genes involved in a wide range of cellular processes and various functions, including transmembrane transporter activity, diversified catalytic activities, protein kinase cascade and HSPs (figure 6*c*). All these processes are involved in the recovery of cellular homeostasis after stress [1,3,45].

Our observation that each HS-responsive miRNA group may have potential targets among all of the groups of stress-responsive genes—the early-up, early-down and late-up genes (figure 6*b*)—most likely resembles the complex kinetics of miRNA-mediated mRNA repression. Importantly, different miRNA groups target genes belonging to the similar GO functional categories (figure 6*c*). This observation may indicate that the same cellular processes that are involved in the formation of the adaptive response to HS may be regulated by HS-responsive miRNAs. This continuous control of cellular processes is probably executed by various miRNAs with different expression levels at different time points after HS to provide fine-tuning of gene expression during the HSR.

It is clear that basic structure of the miRNA : mRNA regulatory network represents a species-specific trait, which can be modulated by different stress stimuli such as HS. It is a challenge to determine whether the interstrain differences in the modulation of this network by HS demonstrated in this study have some adaptive value.

Our analysis of the dynamics of miRNA expression changes occurred on the background of HS-induced suppression of protein synthesis. It is known that the physiological activity of Hsp70 contributes to the rapid recovery of original translational pattern and facilitates the restoration of whole-organism function after HS [23–25,46,47]. We expected that deletion of *hsp70* genes would exert a pronounced effect on the modulation of miRNA expression after HS. However, the modulation of miRNA expression during the HSR appears not to depend on the recovery rate of protein synthesis. Thus, in our studies, we failed to observe the differences in miRNA expression patterns between the closely related *w^1118^* and *hsp70^−^* strains within the first 6 h after HS, when protein synthesis in these strains differs greatly. However, the expression of miRNAs after HS is not Hsp70-independent altogether, because the miRNAs in group #4 (late-down) as well as group #1 in the *hsp70^−^* strain show notable differences in miRNA expression profiles compared with the control strains (figure 4*b*). Thus, we concluded that the absence of Hsp70 affects the expression of miRNAs after HS, which is probably owing to its impact on cellular metabolism. Along these lines, it has recently been shown that overexpression of Hsp70 results in the stabilization of miR-23a levels in an acute lymphoblastic T-cell line, which leads to decreased sensitivity of the cells to heat-induced NOXA-mediated apoptosis [48].

As computational miRNA target prediction shows, *hsp70* transcripts may be a target for regulation by a set of abundant miRNAs; for example, miR-14 and miR-8 (data not shown). It is possible that a tremendous number of *hsp70* transcripts appearing in the cytoplasm after HS can block certain miRNAs (akin to a ‘sponge’ effect) and thereby abrogate the silencing of their authentic targets [49]. Furthermore, recent findings indicate that Hsp70 can directly bind and stabilize ARE-containing mRNAs that are the preferential targets of miRNAs themselves [50]. Therefore, the exact mechanisms underlying the involvement of Hsp70 and other components of the HSR in the miRNA-mediated gene silencing machinery require further investigation by exploring different model species and stress conditions.

## 4. Material and methods

### 4.1. *Drosophila melanogaster* strains

*Drosophila melanogaster* strain *w^1118^* (Df(3R)Hsp70A, Df(3R)Hsp70B), referred to as *hsp70^−^* in which all *hsp70* genes were deleted, was obtained from the Bloomington Drosophila Stock Centre (stock number: 8841). Validation of the absence of *hsp70* genes in the genome of *hsp70^−^* strain was performed, using DNA genotyping, RT-PCR and Western blot. Strains *w^1118^* and *df(1)yw67c23(2)*, referred to as *yw*, were used as controls. It should be noted that *w^1118^* is a closely related strain because it represents one of the parental strains that was explored in the genetic experiments that were conducted to obtain the *hsp70^−^* strain. Another control strain, *yw,* is a common laboratory strain that is unrelated to *w^1118^* and *hsp70^−^* strains. All flies were reared at 25°С on a standard sugar–yeast–agar medium.

### 4.2. Heat treatment

Heat shock protocols used in these experiments were empirically developed. For molecular biological experiments, adult 3- to 5 day old females were used. HS treatment was carried out in a circulating water bath at 38.5°C for 30 min. After 1, 6 and 24 h of recovery after HS at 25°С flies were used for experiments.

For thermotolerance studies, 0–1-day-old flies were anaesthetized and grouped as 25 adult males or females per vial. The next day the flies were exposed to HS, as described above. The survival rate was estimated the next day. Male and female survival did not differ significantly, and hence we grouped the results obtained for both sexes. The sample size for each genotype was 150–200 flies.

### 4.3. Quantitative-PCR

Total RNA was extracted using TRI-reagent (Sigma-Aldrich, USA). After that, total RNA was treated by DNase 1 (Sigma-Aldrich, USA) and used for further analysis. For pri-miRNA, study cDNA was prepared using gene-specific primer and Thermoscript reverse transcriptase (Invitrogen, USA) according to Schmittgen *et al.* [51]. For protein-coding genes analysis, cDNA was prepared, using random primer and MMLV reverse transcriptase (Evrogen, Russia) according to manufacturer's recommendations. Experiment was performed on ABI PRISM 7500 Sequence Detection System (Applied Biosystems, USA). Detection of amplification products was carried out using SYBR Green 1 with the presence of ROX reference dye (Evrogen, Russia) in accordance with the manufacturer's protocol. Specificity of amplified products was verified by sequencing as well as melting curve analysis. Quantification was normalized to ubiquitously expressed *2S* and *snoR442* genes (for pri-miRNAs) and to *rp49* and *αTub* (for protein-coding genes). Calculation of relative expression levels was done, using the equation 2^−ddCt^. The resulting value of the expression for each sample was determined basing on three biological replicates. Sequences of used primers are shown in electronic supplementary material, table S1.

### 4.4. Northern blotting

Typically, 30 µg of total RNA was separated in TBE-based 15% polyacrylamide gel in the presence of 8 M of urea. After electrophoresis, gel fragment containing small RNAs was transferred to Hybond XL (GE Healthcare, UK). The RNA was fixed using EDC cross-linking according to Pall & Hamilton [52]. Prehybridization and hybridization steps were performed by incubating the membrane with 10 ml of ULTRAhyb-Oligo (GE Healthcare, UK). One hundred nanograms of chemically synthesized DNA probe (Evrogen, Russia) were 5′-32P-radiolabelled with polynucleotide kinase (New England Biolabs, USA) and 4 µl γ-32P-ATP (10 mCi ml^−1^) and purified, using an Illustra Microspin G-25 column (GE Healthcare, UK). Membranes were analysed by phosphorimaging system (Perkin Elmer, USA). Sequences of used probes are shown in electronic supplementary material, table S1.

### 4.5. Western blotting

Protein extracts of flies exposed to HS were obtained at specified time intervals (1, 3, 6 and 24 h) after HS. Control flies were kept at 25°C. After electrophoresis, proteins were transferred to a nitrocellulose membrane (GE Healthcare, UK). Hsp70 was detected using monoclonal antibodies 7FB that are specific for the inducible Hsp70. Antibodies were kindly provided by Prof. S. Lindquist. The anti-Ago1 (ab5070, 1/750) antibodies used were purchased from Abcam, USA. Detection of a ubiquitous *Drosophila* protein, actin 5c, was used as a loading control exploring the antibody to actin, clone c4 (Millipore, USA). Immune complexes were detected, using chemiluminescence (ECL Kit, GE Healthcare, UK) on a ChemiDoc MP system (Bio-Rad, USA) after incubation with secondary antibodies conjugated with horseradish peroxidase (Millipore, USA).

### 4.6. Incorporation of [S35]-labelled methionine into *Drosophila melanogaster* salivary gland proteins

The third-instar larvae were subjected to HS for 30 min at 38.5°C. At intervals of 1, 3 and 6 h after HS, salivary glands were dissected and incubated for 40 min at 25°C in 20 µl of Schneider's insect medium lacking methionine (Sigma-Aldrich, USA) and supplemented with 1 µl (1 µCi) of [35S]-labelled methionine (GE Healthcare, UK). Salivary glands from the larvae were kept at 25°C and used as the control. Labelled salivary glands were lysed in 20 µl of Laemmli sample buffer. Protein extracts were separated in a 10% SDS–polyacrylamide gel. Equal amounts of each sample were used. Incorporation of the radioactive label was evaluated, using radioautographic exposure.

### 4.7. Small RNA libraries preparation

Total RNA was isolated using Tri-reagent (Sigma-Aldrich). Approximately 25 µg of total RNA was separated, using 15% polyacrylamide gel electrophoresis containing 8 M urea. After incubation in an ethidium bromide solution (0.5 µg ml^−1^), gel fragments corresponding to the small RNAs fraction were excised. We used chemically synthesized RNAs (Syntol, Russia) corresponding to 21 and 27 nucleotides as size markers.

Cloning of small RNA libraries was conducted, using the Illumina TruSeq Small RNA Prep Kit (Illumina, USA) according to the manufacturer's protocol. Each sample was made in two biological replicates except for the *yw* 24 h sample, which is represented only once. Sequencing of the small RNAs was performed on an Illumina HiSeq 2000 (Illumina).

### 4.8. Statistical analysis

Thermotolerance results were analysed using the Mann–Whitney test. Student's *t*-test and the Mann–Whitney test were used to compare mRNA levels between the studied groups at specified time intervals. *p*-values ≤ 0.05 were considered to be statistically significant.

### 4.9. Bioinformatic analysis

Pre-processing and mapping of small RNA libraries to the *dm3 Drosophila* genome assembly was performed as previously described [27]. The number of reads mapped to the miRNAs was counted, using the BedTools toolkit (v. 2.22) and miRBase (r.19) annotation [53].

miRNAs that were differentially expressed after HS exposure were evaluated using two approaches. First, we performed the pairwise comparison of the studied strains at each time point (e.g. control versus control, 1 versus 1 h, etc.). This comparison was performed using edgeR (v. 3.10.2) and DESeq (v. 1.22) packages in an R environment (v. 3.2.2) [54,55]. The miRNA was considered to be differentially expressed whether there were more than or equal to 50 counts and whether it was detected by both programmes with *p*_adj_ ≤ 0.05 (BH correction) and log_2_ FC ≥ 1.5. RNA editing analysis was performed with the REDItools software suite in de novo calling mode [56].

Second, to identify miRNAs with significant temporal expression changes and differences between the strains, we performed a time-course analysis of miRNA expression profiles. For this, counts of the reads for each miRNA were converted to RPM, log_2_-transformed and z-scaled across the expression profiles. The normalized expression values at each time point were additionally normalized to the corresponding expression value at the control time point before HS such that the expression of each miRNA at control point was 0. Time-course analysis was evaluated, using the maSigPro package (1.40.0). The parameters used were a global regression to find differentially expressed genes with a *Q*-value = 0.05 (BH correction), a stepwise regression to find significantly changed expression profiles with the ‘backward’ method, and a cut-off value of *α* = 0.05 [57]. The procedure of model fitting was performed three times, using each of the strains as a reference. miRNAs with a regression model of *R*^2^ ≥ 0.6 were considered as miRNAs with a significantly changed expression profile. Identified miRNAs were combined to identify differentially expressed miRNAs at least in one strain, two strains or in all three strains. For the hierarchical clustering of miRNAs, the *Y^S1^* distance and ‘complete’ methods were applied to miRNAs with significantly different expression profiles in at least two strains. In our analysis, the *Y^S1^* distance was chosen, because according to the last published benchmark, this metric outperforms the other types of correlation-based distances [58,59].

To identify the functions of all levelled miRNAs targets (class #1 miRNAs; for a description of the defined miRNA classes, see the text), we used targets predicted either by TargetScanFly v. 6.2 or DIANA microT v. 4 [60,61]. Only targets that were unique for a given miRNA group across all of the groups were kept. For targeting analysis of the stress-responsive genes, we used three additional prediction algorithms: the Miranda-SVRscore, PicTarFly and MicroCosm databases [62–64]. Only targets predicted by at least two of these algorithms were considered.

The analysis of the enriched GO terms was performed using the topGO package (2.22.0) with the *elim* algorithm and a *p*-value = 0.05 (Fisher's exact test) and *Q*-value = 0.05 (BH correction) as cut-offs. All of the terms with evidence of IEA, RCA and ND were removed before enrichment analysis. Redundant GO terms were removed, using REVIGO software [65]. The semantic similarity of the enriched GO terms (*p*-value ≤ 0.05) of groups of miRNAs targets was evaluated with the GOSemSim package (1.28.1) [66]. To estimate the *p*-value of the given similarity score, we calculated the semantic similarity of randomly sampled set pairs of GO terms of the same size; the *p*-value was estimated as the frequency of random GO term group pairs having with the same or greater semantic similarity score (*n* = 500).

## Supplementary Material

Figure S1

## Supplementary Material

Figure S2

## Supplementary Material

Figure S3

## Supplementary Material

Figure S4

## Supplementary Material

Supplementary Tables S1–S5

## Data accessibility

Extended data are provided in the supplementary material (tables S1–S5 and figures S1–S4). Small RNA sequencing data are deposited at Gene Expression Omnibus (accession no. GSE76844).
