# Supplementary figures and images for: Interplay between RNA interference and heat shock response systems in Drosophila melanogaster
Source: Open Biol. 2016 Oct 19;6(10):160224. doi: 10.1098/rsob.160224 (PMC5090062; doi:10.1098/rsob.160224)

A

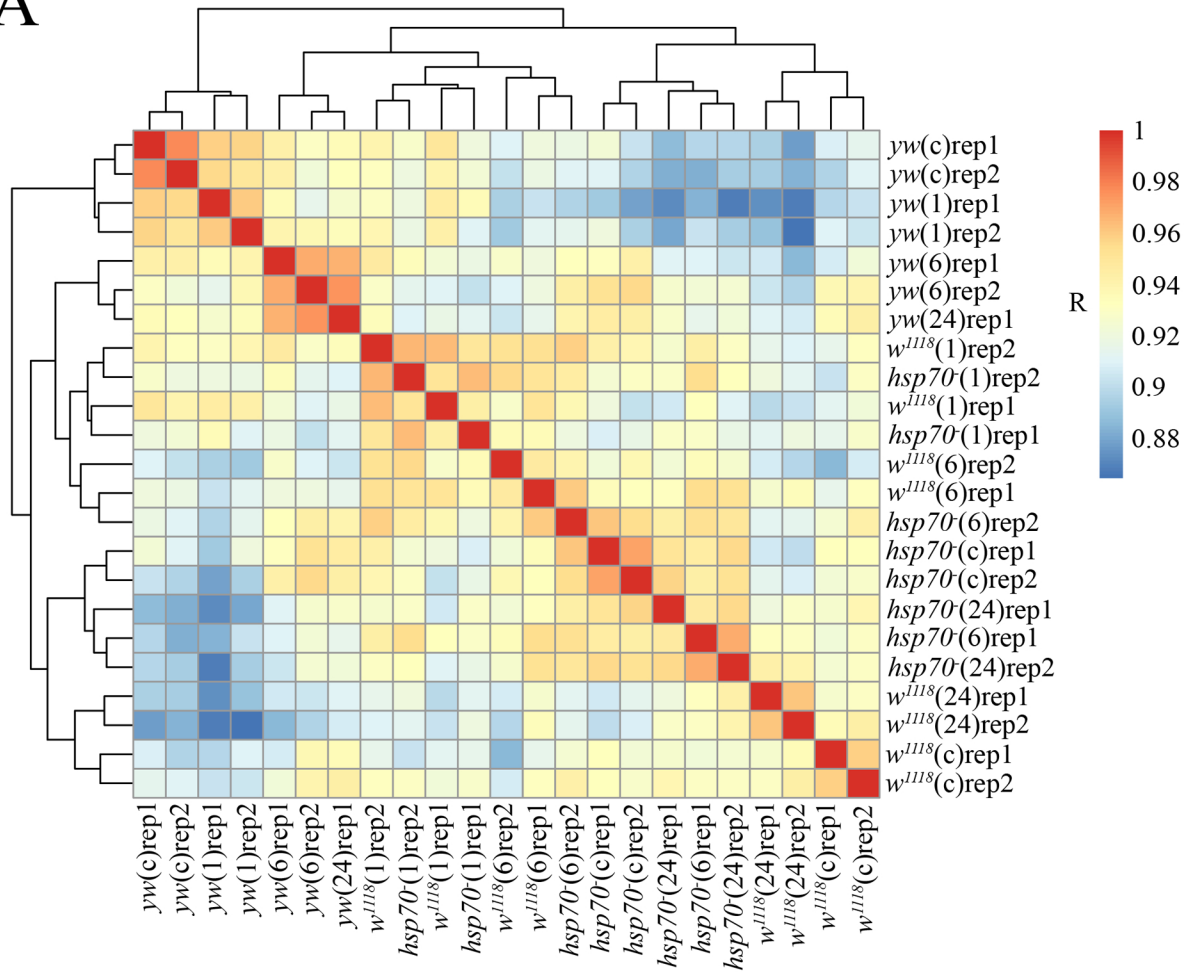

B

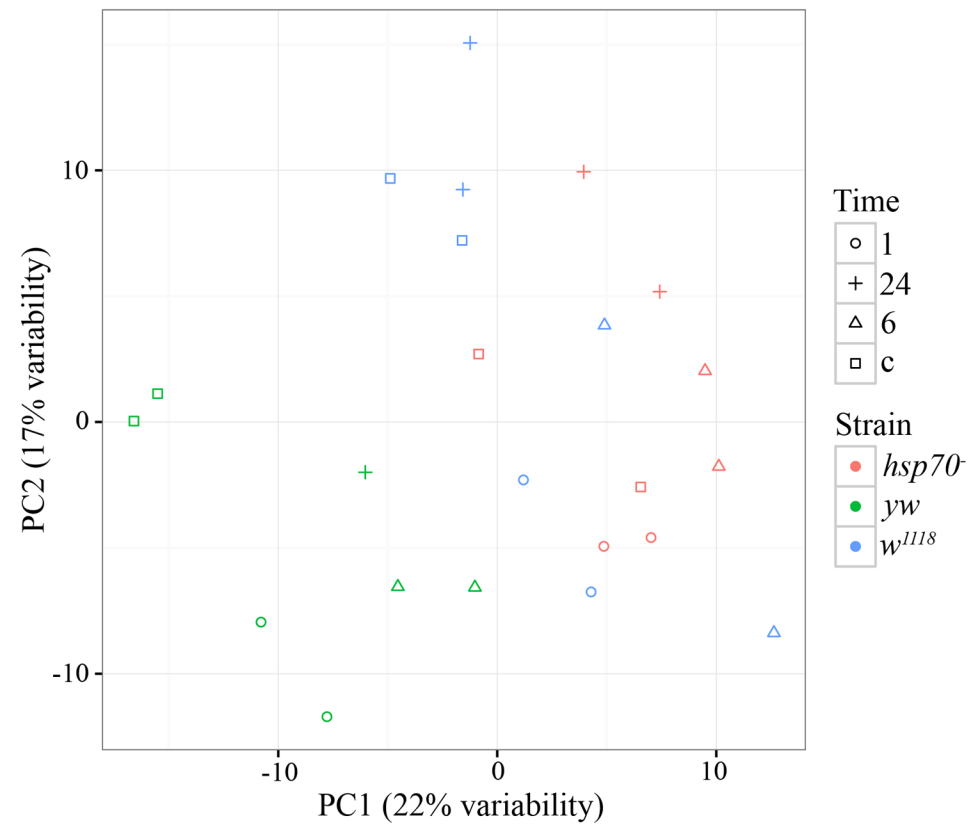

Supplement: Figure S1 [file rsob160224supp1.pdf]

A

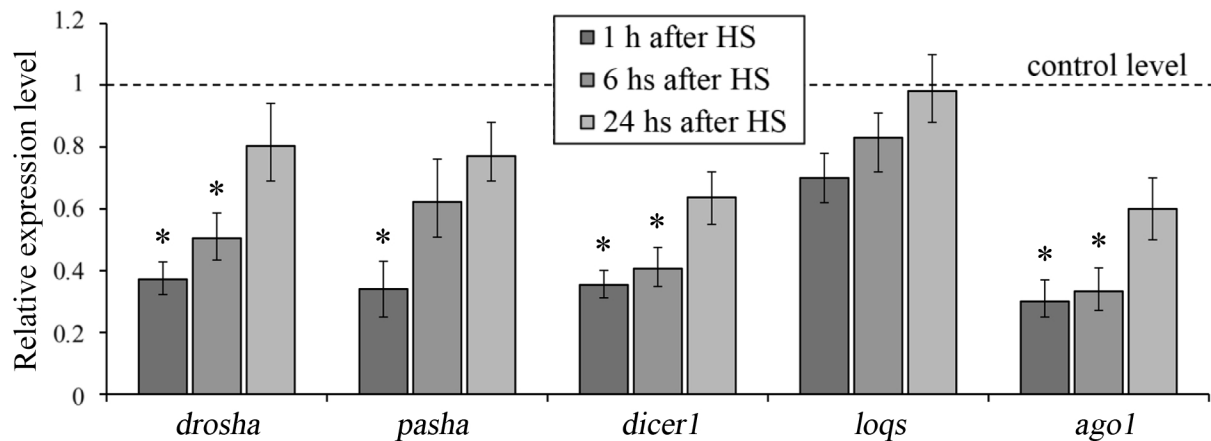

B

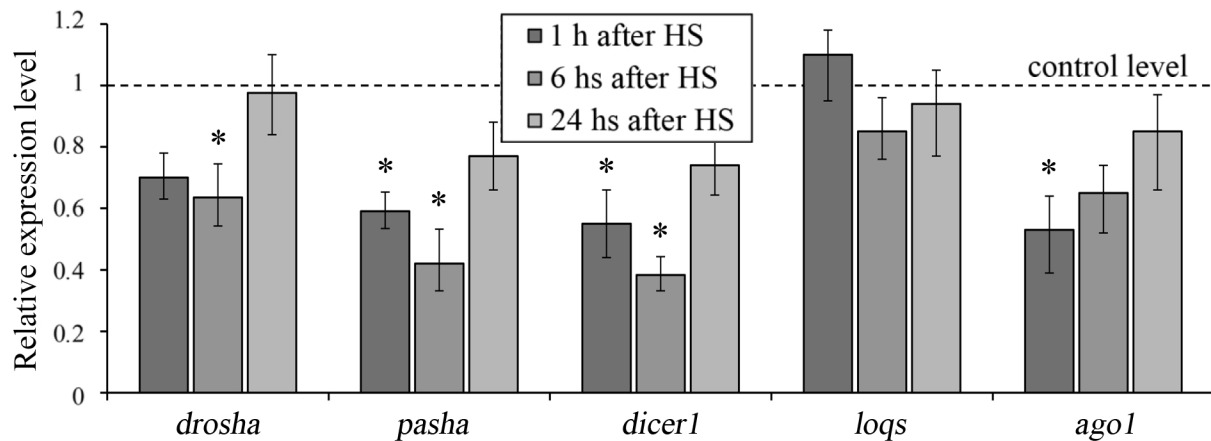

Supplement: Figure S4 [file rsob160224supp4.pdf]
