# Supplementary material for: Interplay between RNA interference and heat shock response systems in Drosophila melanogaster
Source: Open Biol. 2016 Oct 19;6(10):160224. doi: 10.1098/rsob.160224 (PMC5090062; doi:10.1098/rsob.160224)
Supplement: Figure S2 [file rsob160224supp2.pdf]

A

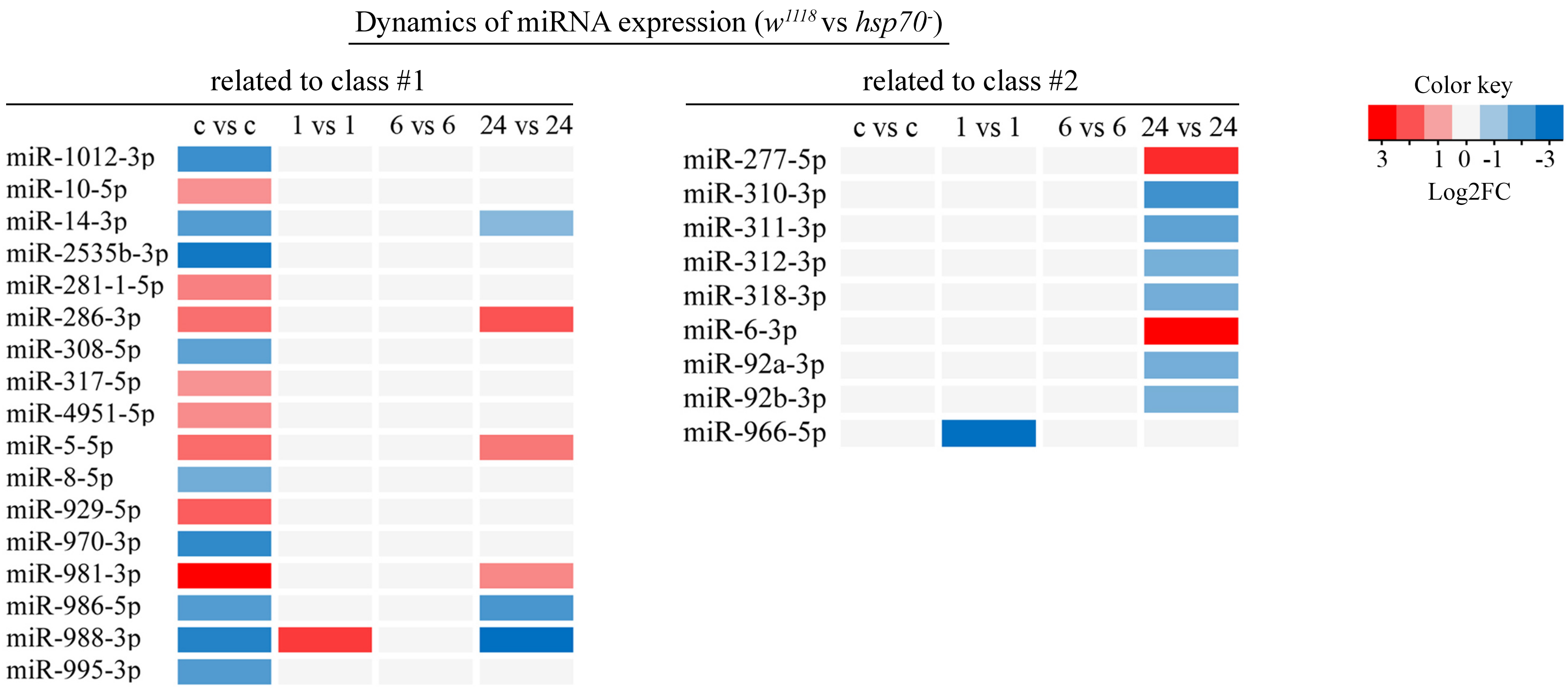

Dynamics of miRNA expression (w^{1118} vs $hsp70^{-}$)
related to class #1
c vs c
1 vs 1
6 vs 6
24 vs 24
miR-1012-3p
miR-10-5p
miR-14-3p
miR-2535b-3p
miR-281-1-5p
miR-286-3p
miR-308-5p
miR-317-5p
miR-4951-5p
miR-5-5p
miR-8-5p
miR-929-5p
miR-970-3p
miR-981-3p
miR-986-5p
miR-988-3p
miR-995-3p
related to class #2
c vs c
1 vs 1
6 vs 6
24 vs 24
miR-277-5p
miR-310-3p
miR-311-3p
miR-312-3p
miR-318-3p
miR-6-3p
miR-92a-3p
miR-92b-3p
miR-966-5p
Color key
3
1
0
-1
-3
Log2FC

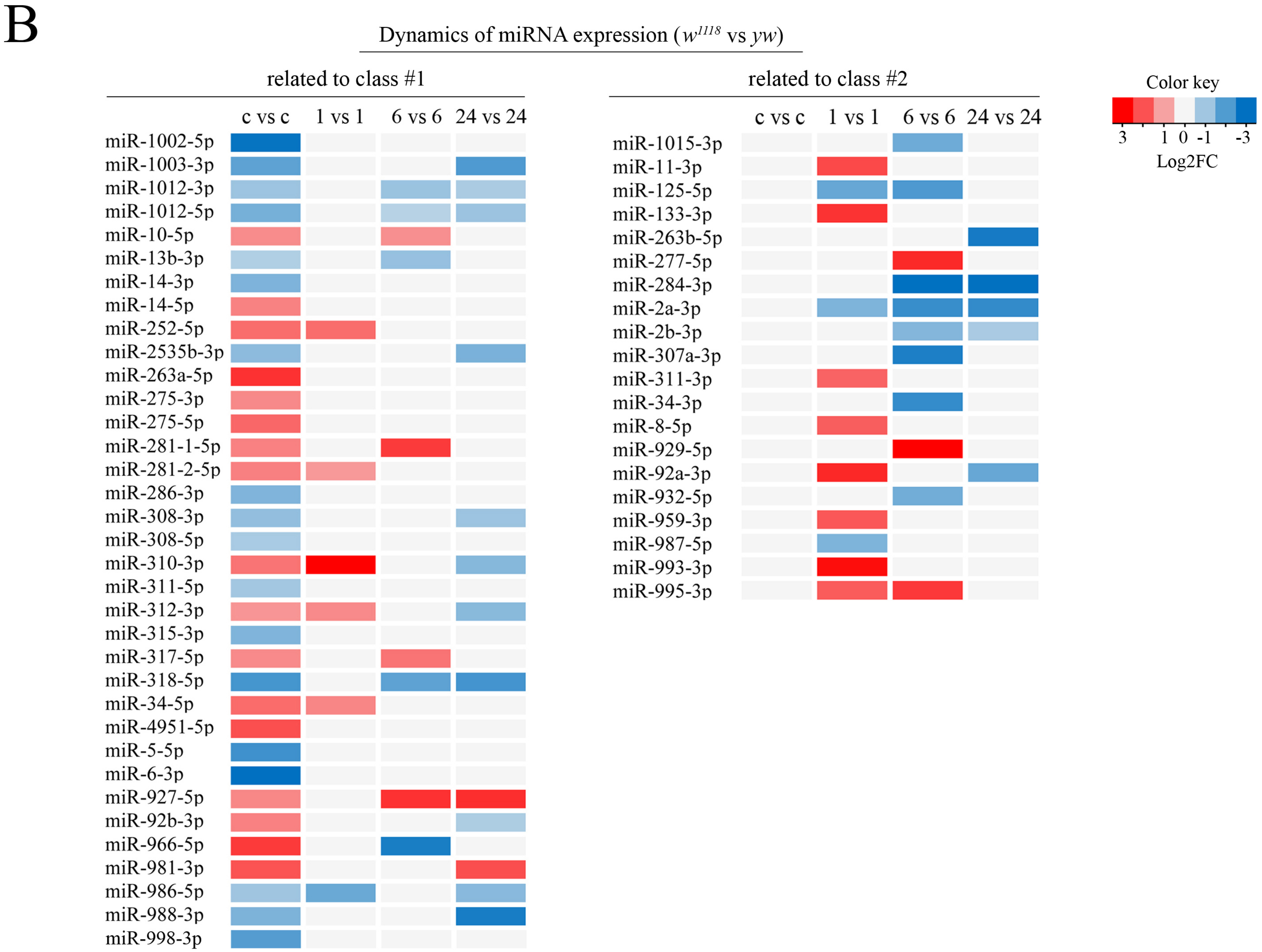
B
Dynamics of miRNA expression (w1118 vs yw)
related to class #1
c vs c
1 vs 1
6 vs 6
24 vs 24
miR-1002-5p
miR-1003-3p
miR-1012-3p
miR-1012-5p
miR-10-5p
miR-13b-3p
miR-14-3p
miR-14-5p
miR-252-5p
miR-2535b-3p
miR-263a-5p
miR-275-3p
miR-275-5p
miR-281-1-5p
miR-281-2-5p
miR-286-3p
miR-308-3p
miR-308-5p
miR-310-3p
miR-311-5p
miR-312-3p
miR-315-3p
miR-317-5p
miR-318-5p
miR-34-5p
miR-4951-5p
miR-5-5p
miR-6-3p
miR-927-5p
miR-92b-3p
miR-966-5p
miR-981-3p
miR-986-5p
miR-988-3p
miR-998-3p
related to class #2
c vs c
1 vs 1
6 vs 6
24 vs 24
miR-1015-3p
miR-11-3p
miR-125-5p
miR-133-3p
miR-263b-5p
miR-277-5p
miR-284-3p
miR-2a-3p
miR-2b-3p
miR-307a-3p
miR-311-3p
miR-34-3p
miR-8-5p
miR-929-5p
miR-92a-3p
miR-932-5p
miR-959-3p
miR-987-5p
miR-993-3p
miR-995-3p
Color key
3
1
0
-1
-3
Log2FC

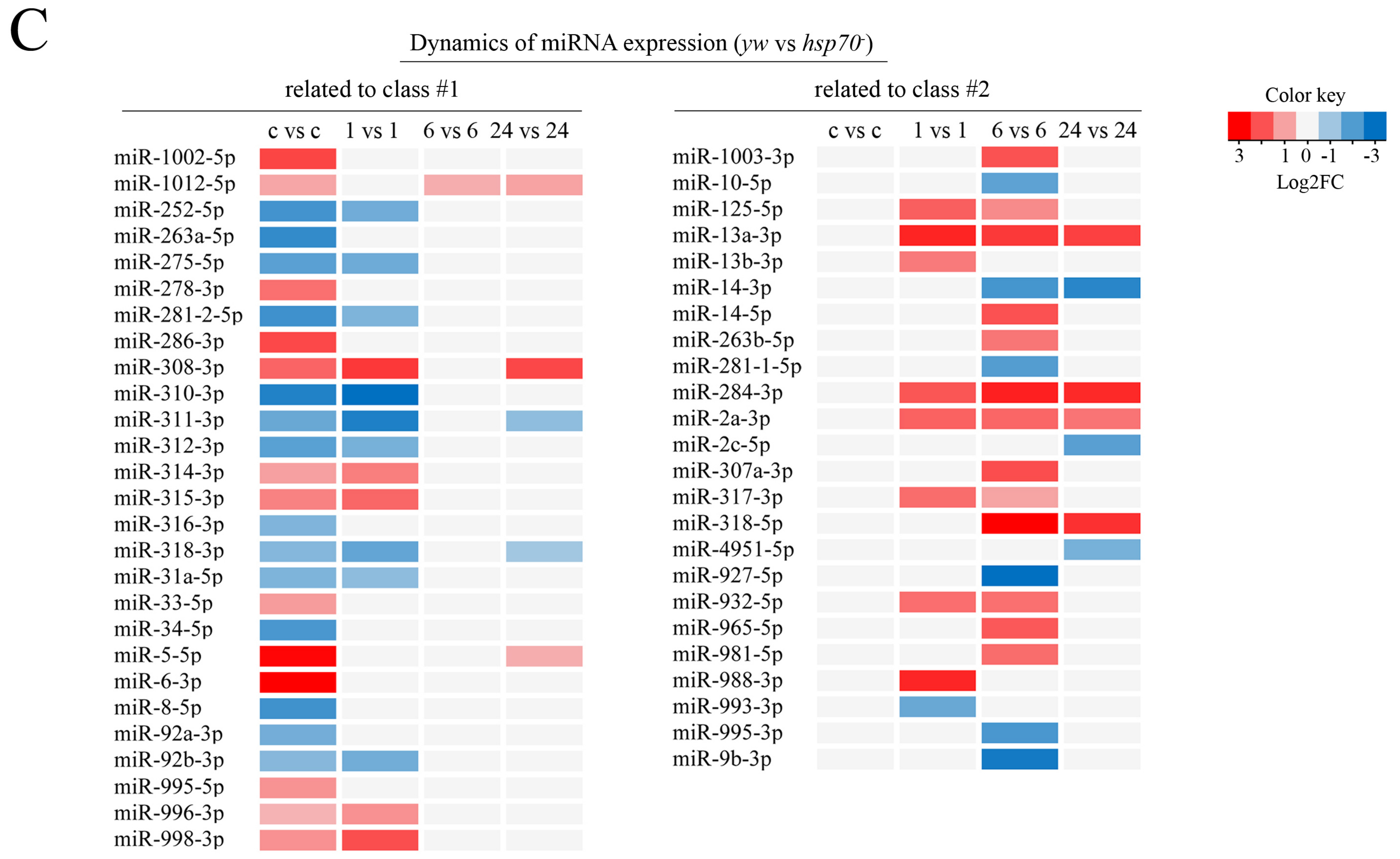
C
Dynamics of miRNA expression (yw vs hsp70-)
related to class #1
c vs c
1 vs 1
6 vs 6
24 vs 24
miR-1002-5p
miR-1012-5p
miR-252-5p
miR-263a-5p
miR-275-5p
miR-278-3p
miR-281-2-5p
miR-286-3p
miR-308-3p
miR-310-3p
miR-311-3p
miR-312-3p
miR-314-3p
miR-315-3p
miR-316-3p
miR-318-3p
miR-31a-5p
miR-33-5p
miR-34-5p
miR-5-5p
miR-6-3p
miR-8-5p
miR-92a-3p
miR-92b-3p
miR-995-5p
miR-996-3p
miR-998-3p
related to class #2
c vs c
1 vs 1
6 vs 6
24 vs 24
miR-1003-3p
miR-10-5p
miR-125-5p
miR-13a-3p
miR-13b-3p
miR-14-3p
miR-14-5p
miR-263b-5p
miR-281-1-5p
miR-284-3p
miR-2a-3p
miR-2c-5p
miR-307a-3p
miR-317-3p
miR-318-5p
miR-4951-5p
miR-927-5p
miR-932-5p
miR-965-5p
miR-981-5p
miR-988-3p
miR-993-3p
miR-995-3p
miR-9b-3p
Color key
3
1
0
-1
-3
Log2FC
