# Supplementary material for: Interplay between RNA interference and heat shock response systems in Drosophila melanogaster
Source: Open Biol. 2016 Oct 19;6(10):160224. doi: 10.1098/rsob.160224 (PMC5090062; doi:10.1098/rsob.160224)

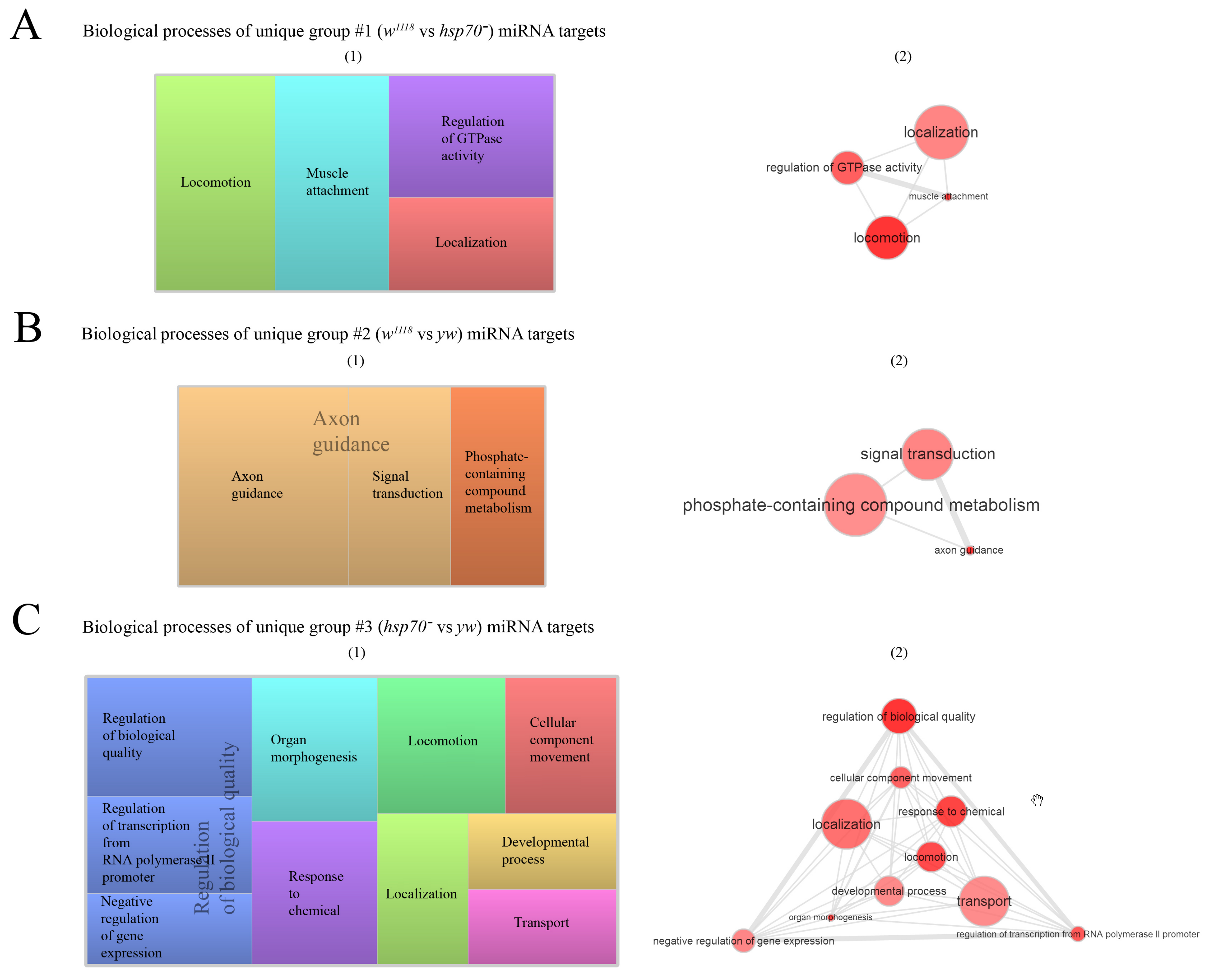

A
Biological processes of unique group #1 (w^{1118} vs $hsp70^{-}$) miRNA targets
(1)
Locomotion
Muscle attachment
Regulation of GTPase activity
Localization
(2)
localization
regulation of GTPase activity
muscle attachment
locomotion
B
Biological processes of unique group #2 (w^{1118} vs yw) miRNA targets
(1)
Axon guidance
Axon guidance
Signal transduction
Phosphate-containing compound metabolism
(2)
signal transduction
phosphate-containing compound metabolism
axon guidance
C
Biological processes of unique group #3 ($hsp70^{-}$ vs yw) miRNA targets
(1)
Regulation of biological quality
Regulation of biological quality
Regulation of transcription from RNA polymerase II promoter
Negative regulation of gene expression
Organ morphogenesis
Response to chemical
Locomotion
Cellular component movement
Localization
Developmental process
Transport
(2)
regulation of biological quality
cellular component movement
response to chemical
localization
locomotion
developmental process
transport
organ morphogenesis
negative regulation of gene expression
regulation of transcription from RNA polymerase II promoter

D

Biological processes of shared group #1 (w^{1118} vs *hsp70*$^{-}$ & w^{1118} vs *yw*) miRNA targets

(1) (2)

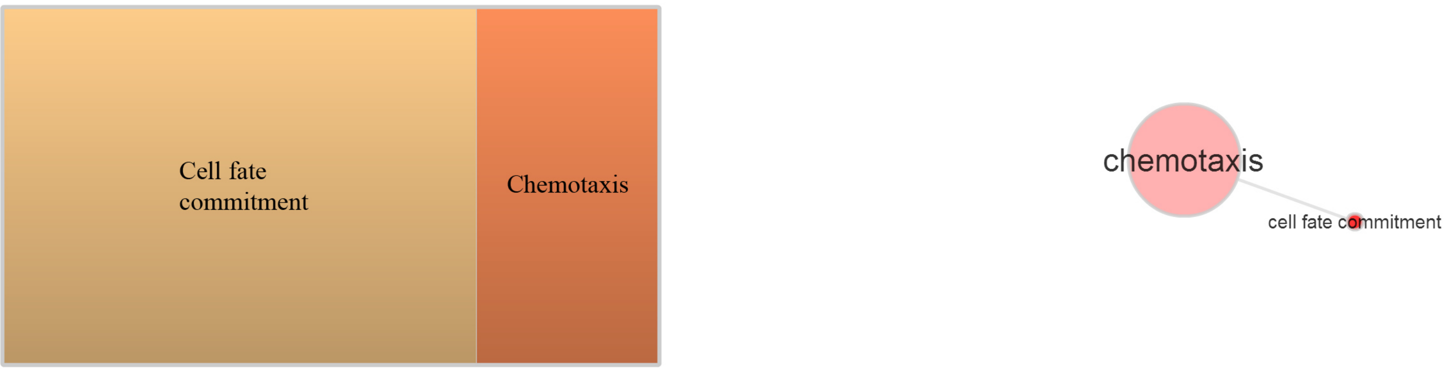

E

Biological processes of shared group #2 (w^{1118} vs *yw* & *hsp70*$^{-}$ vs *yw*) miRNA targets

(1) (2)

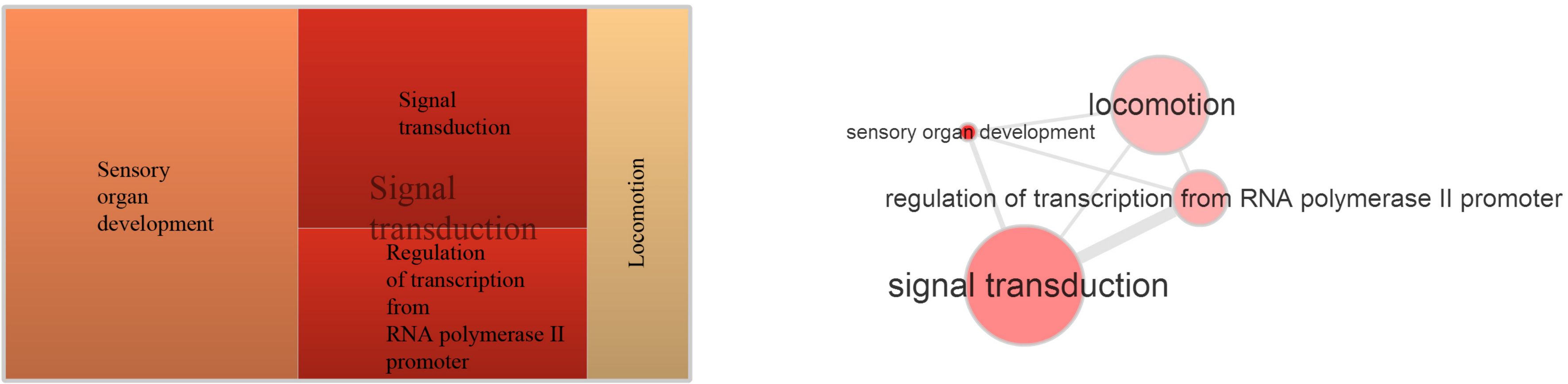

F

Biological processes of shared group #3 (w^{1118} vs *hsp70*$^{-}$ & *hsp70*$^{-}$ vs *yw*) miRNA targets

(1) (2)

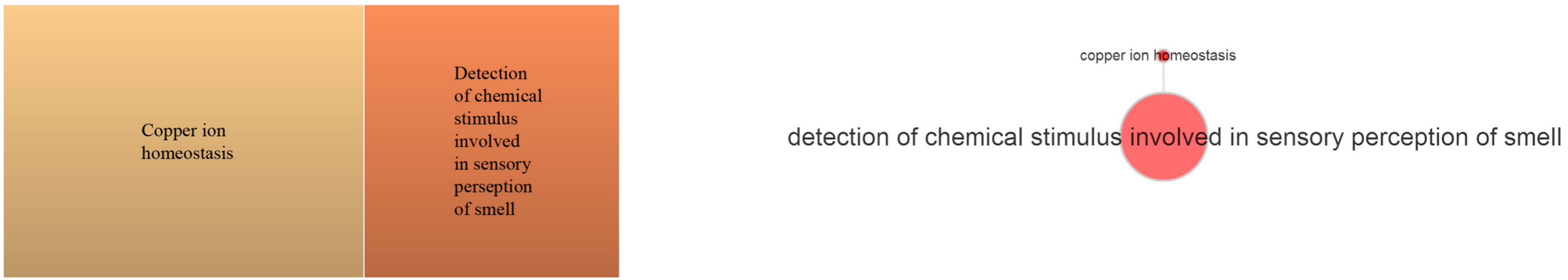

G

Biological processes of common miRNA targets

(1)

(2)

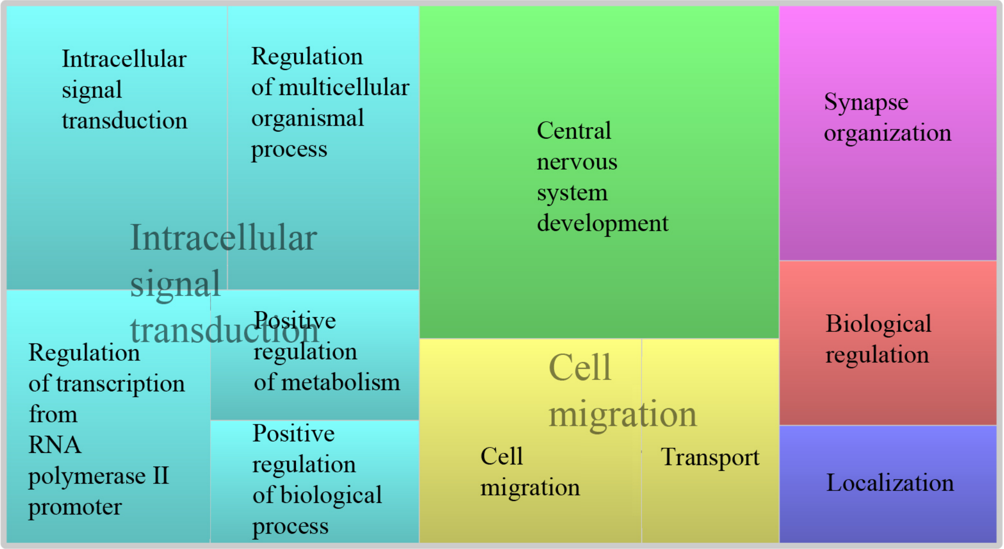

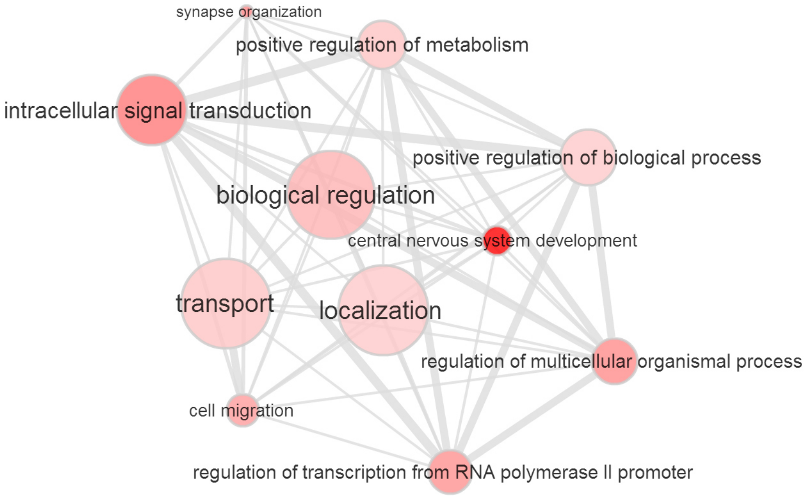

Supplement: Figure S3 [file rsob160224supp3.pdf]
